# Editorial: Stereotactic radioablation of cardiac arrhythmias: pros and cons

**DOI:** 10.3389/fcvm.2023.1208851

**Published:** 2023-06-20

**Authors:** Maria Lucia Narducci, Francesco Cellini, Andrea Natale

**Affiliations:** ^1^ Cardiovascular Science Department, Agostino Gemelli University Polyclinic, IRCCS, Rome, Italy; ^2^ Dipartimento Universitario Diagnostica per Immagini, Radioterapia Oncologica ed Ematologia Università Cattolica del Sacro Cuore, Roma, Italy; ^3^ Texas Cardiac Arrhythmia Institute, Center for Atrial Fibrillation, St. David’s Medical Center, Austin, TX, United States

**Keywords:** ablation < electrophysiology, ventricular tachycardia, stereotactic ablation body radiation therapy, stereotactic ablation, atrial fibrillation

**Editorial on the Research Topic** Cardiac arrhythmias and stereotactic radioblation: pros and cons

Ventricular tachyarrhythmias (VT) represent a life-threatening condition often observed in patients with structural heart disease, with consequent serious impact to patient survival and quality of life. The most common cause of recurrent monomorphic VT is the presence of an electroanatomic scar and related re-entry mechanisms. Particularly, radiofrequency catheter ablation (RFA) represents the gold standard for scar-related VT ablation, along with optimal medical therapy. The localization of an arrhythmic substrate inaccessible using catheter-based ablation techniques, usually due to a location deep on the endocardial or epicardial surfaces of the myocardium, is the most common cause of RFA failure (1). With regard to this, noninvasive stereotactic arrhythmia radioablation (STAR) uses stereotactic body radioablation therapy (SBRT) as a novel treatment modality for refractory VT (2, 3). Stereotactic body radioablation therapy delivers high-dose focused radiation in a single fraction of 25 Gy, allowing ablation through induction of myocardial scarring and a second mechanism related to reprogramming of electrical conduction (4). The procedure is completely noninvasive; therefore, it can be performed in patients with contraindications to invasive ablation procedures. Cardiac STAR should be performed at experienced centers, preferably within clinical trials, in cooperation between cardiac electrophysiologists and radiation oncologists and physicist. In this Research Topic of Frontiers in Cardiovascular Medicine, we aimed to report on three different themes such as:

- comprehensive review of the literature on STAR
- complex case reports, original research, and new studies on VT STAR
- new frontiers: STAR and atrial fibrillation (AF)

## Comprehensive review of literature on STAR

In the first systematic review, Volpato et al. provided an overview of the available studies on VT STAR, describing the potential indications and technical aspects of this promising therapy. Particularly, STAR can be considered a true treatment for patients with structural heart disease who have recurrent VT or electrical storm despite optimal antiarrhythmic drug therapy and prior catheter RFA, or in case of contraindications to RFA, such as in the case of mechanical aortic and mitral prosthetic valves. The purpose of the second systematic review by Franzetti et al. is to collect available evidence on the feasibility and efficacy of STAR in the treatment of AF. Particular attention should be paid to the safety rather than the efficacy of STAR, given the benign nature of AF. Uncertainties remain, especially regarding the definition of the treatment plan and the role of the target motion. In this setting, more information about the toxicity profile of this new approach is compulsory before applying STAR to AF in clinical practice.

## Complex case reports, original research and new trials on VT STAR

In this Research Topic, we designed the observational study “VT-Art Consortium” in order to provide insight into the efficacy and safety of STAR through a matched pair analysis, in two groups of patients with VT undergoing radiation therapy versus conventional ablation.

Particularly, the early response to STAR may be unpredictable and probably does not reflect the final outcome of irradiation as demonstrated by preliminary results from the SMART VT trial using the volumetric modulated arc therapy technique and three 6 MeV flattening filter-free photon beam fields. Functional changes could appear relatively early, manifesting as a rapide decrease of VT burden, as well as transient exacerbation of the arrhythmia. The SMART-VT study is ongoing, and the clinical course of the two presented cases clearly indicates that the toxicity profile of the STAR can only be assessed as part of a comprehensive clinical trial.

Wight et al. analyzed long-term follow-up of STAR for refractory VT in advanced heart failure patients, with evidence of an immediate reduction in VT burden after treatment as an important bridge to transplantion in this particular clinical setting. We have also published a challenging case series on the feasibility of repeated STAR in recurrent VT, with good acute and mid-term safety.

Four case reports were included in our Research Topic: successful VT STAR in two complex cases, such as patient with pleurodesis and patient with multiple devices (valve prosthesis, biventricular defibrillator and contractility modulation device); histopathological examination of the irradiated ventricle with evidence of multifocal mosaic-like fibrosis; feasibility of ultrasound guidance with probe in parasternal viewing position during treatment. On this regard, in the single center study by Casula et al., a prototype of an automatic ultrasonographic imaging acquisition system was developed using an artificial intelligence algorithm to calculate cardiac displacement in real-time. In addition, Dvorak et al. proposed a new technique for geometry deformation margin with Cyberknife before STAR for better control of the risk of “target underdose”.

## New frontiers: STAR and atrial fibrillation

A prospective phase-II trial was designed to evaluate the safety of LINAC-based STAR (ClinicalTrials.gov: NCT04575662). Di Monaco et al. selected 5 elderly patients with refractory AF undergoing STAR, without evidence of acute toxicity.

## Future directions

Non-invasive ablation of cardiac arrhythmias with STAR is generating considerable enthusiasm as an emerging treatment modality for VT. We consider this Research Topic a unique opportunity to share different views on this innovative treatment in electrophysiology. Current experience does not support the view that STAR can replace conventional catheter VT ablation. Larger prospective studies and randomized trials are needed to evaluate the efficacy and the long-term safety of this new treatment. Furthermore, considering STAR as an emerging treatment modality in heart failure patients undergoing heart transplantion, histopathological and molecular study could provide important data for the development of an accurate biological model of the antiarrhythmic effect of STAR.
